# Supplementary material for: Causal Relationship between Aging and Anorexia Nervosa: A White-Matter-Microstructure-Mediated Mendelian Randomization Analysis
Source: Biomedicines. 2024 Aug 16;12(8):1874. doi: 10.3390/biomedicines12081874 (PMC11351342; doi:10.3390/biomedicines12081874)
Supplement: Supplementary file 1 [file biomedicines-12-01874-s001.zip › Supplementary Figure/Figure S4/Singe SNP plot of MR analysis for TL to ubm-b-1806.pdf]

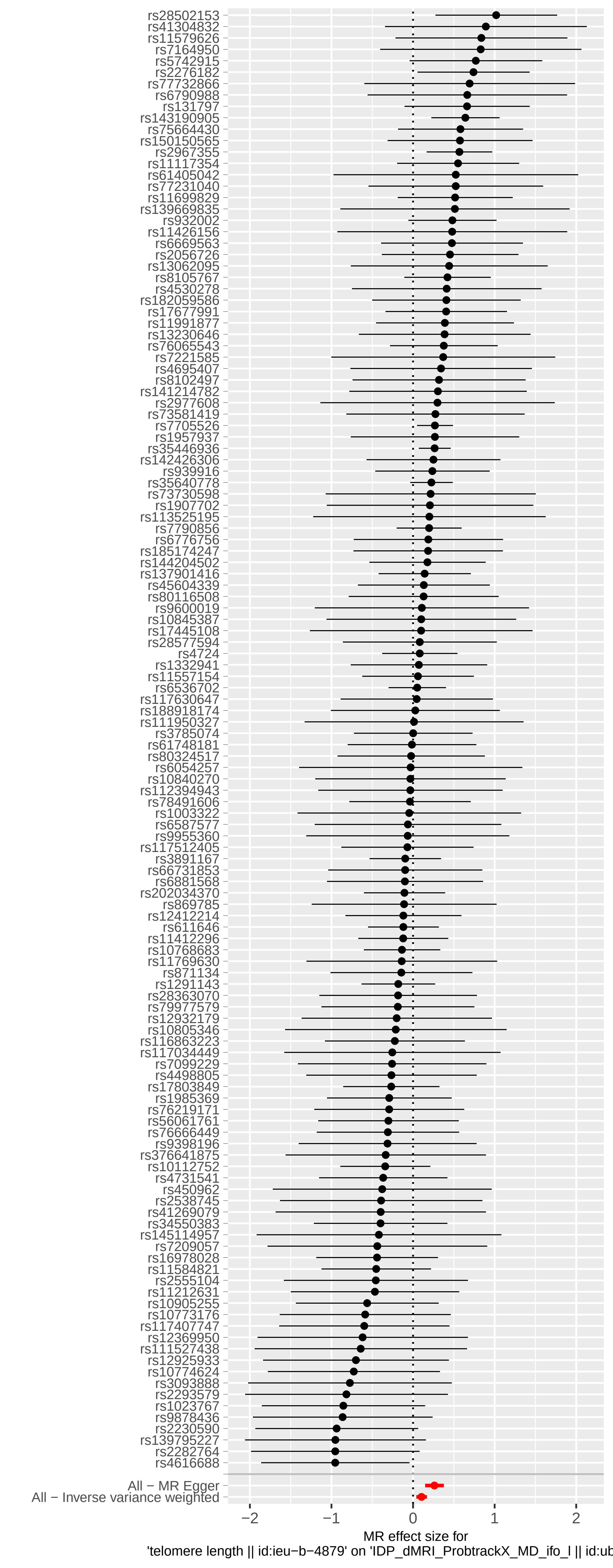
rs28502153
rs41304832
rs11579626
rs7164950
rs5742915
rs2276182
rs77732866
rs6790988
rs131797
rs143190905
rs75664430
rs150150565
rs2967355
rs11117354
rs61405042
rs77231040
rs11699829
rs139669835
rs932002
rs11426156
rs6669563
rs2056726
rs13062095
rs8105767
rs4530278
rs182059586
rs17677991
rs11991877
rs13230646
rs76065543
rs7221585
rs4695407
rs8102497
rs141214782
rs2977608
rs73581419
rs7705526
rs1957937
rs35446936
rs142426306
rs939916
rs35640778
rs73730598
rs1907702
rs113525195
rs7790856
rs6776756
rs185174247
rs144204502
rs137901416
rs45604339
rs80116508
rs9600019
rs10845387
rs17445108
rs28577594
rs4724
rs1332941
rs11557154
rs6536702
rs117630647
rs188918174
rs111950327
rs3785074
rs61748181
rs80324517
rs6054257
rs10840270
rs112394943
rs78491606
rs1003322
rs6587577
rs9955360
rs117512405
rs3891167
rs66731853
rs6881568
rs202034370
rs869785
rs12412214
rs611646
rs11412296
rs10768683
rs11769630
rs871134
rs1291143
rs28363070
rs79977579
rs12932179
rs10805346
rs116863223
rs117034449
rs7099229
rs4498805
rs17803849
rs1985369
rs76219171
rs56061761
rs76666449
rs9398196
rs376641875
rs10112752
rs4731541
rs450962
rs2538745
rs41269079
rs34550383
rs145114957
rs7209057
rs16978028
rs11584821
rs2555104
rs11212631
rs10905255
rs10773176
rs117407747
rs12369950
rs111527438
rs12925933
rs10774624
rs3093888
rs2293579
rs1023767
rs9878436
rs2230590
rs139795227
rs2282764
rs4616688
All − MR Egger
All − Inverse variance weighted
−2
−1
0
1
2
MR effect size for
'telomere length || id:ieu−b−4879' on 'IDP_dMRI_ProbtrackX_MD_ifo_l || id:ub
